# Short physical performance battery as a practical tool to assess mortality risk in chronic obstructive pulmonary disease

**DOI:** 10.1093/ageing/afaa138

**Published:** 2020-09-07

**Authors:** Jilles M Fermont, Divya Mohan, Marie Fisk, Charlotte E Bolton, William Macnee, John R Cockcroft, Carmel McEniery, Jonathan Fuld, Joseph Cheriyan, Ruth Tal-Singer, Hana Müllerova, Angela M Wood, Ian B Wilkinson, Michael I Polkey, the ERICA consortium

**Affiliations:** 1 Division of Experimental Medicine and Immunotherapeutics, Department of Medicine, University of Cambridge, Cambridge, UK; 2 British Heart Foundation Cardiovascular Epidemiology Unit, Department of Public Health and Primary Care, University of Cambridge, Cambridge, UK; 3 Medical Innovation, Value Evidence and Outcomes, GSK, Collegeville, PA, USA; 4 Division of Respiratory Medicine and NIHR Nottingham BRC Respiratory Theme, University of Nottingham, Nottingham, UK; 5 Centre for Inflammation Research, Queen’s Medical Research Institute, University of Edinburgh, Edinburgh, UK; 6 Department of Cardiology, Columbia University Medical Centre, New York, NY, USA; 7 Department of Respiratory Medicine, Cambridge University Hospitals NHS Foundation Trust, Cambridge, UK; 8 GSK R&D, Uxbridge, UK; 9 British Heart Foundation Centre of Research Excellence, University of Cambridge, Cambridge, UK; 10 National Institute for Health Research Blood and Transplant Research Unit in Donor Health and Genomics, University of Cambridge, Cambridge, UK; 11 National Institute for Health Research Cambridge Biomedical Research Centre, University of Cambridge and Cambridge University Hospitals, Cambridge, UK; 12 Health Data Research UK Cambridge, Wellcome Genome Campus and University of Cambridge, Cambridge, UK; 13 Medical Research Council Biostatistics Unit, Cambridge Institute of Public Health, University of Cambridge, Cambridge, UK; 14 Alan Turing Institute, London, UK; 15 Cambridge Clinical Trials Unit, Cambridge University Hospitals NHS Foundation Trust, Addenbrooke’s Hospital, Cambridge, UK; 16 Department of Respiratory Medicine, Royal Brompton Hospital, London, UK

**Keywords:** chronic obstructive pulmonary disease, mortality, biomarkers, skeletal muscle, older people

## Abstract

**Rationale:**

chronic obstructive pulmonary disease (COPD) is a leading cause of mortality and common in older adults. The BODE Index is the most recognised mortality risk score in COPD but includes a 6-minute walk test (6MWT) that is seldom available in practise; the BODE Index may be better adopted if the 6MWT was replaced.

**Objectives:**

we investigated whether a modified BODE Index in which 6MWT was replaced by an alternative measure of physical capacity, specifically the short physical performance battery (SPPB) or components, retained its predictive ability for mortality in individuals with COPD.

**Methods:**

we analysed 630 COPD patients from the ERICA cohort study for whom UK Office for National Statistics verified mortality data were available. Variables tested at baseline included spirometry, 6MWT, SPPB and its components (4-m gait speed test [4MGS], chair stand and balance). Predictive models were developed using stratified multivariable Cox regression, and assessed by C-indices and calibration plots with 10-fold cross-validation and replication.

**Results:**

during median 2 years of follow-up, 60 (10%) individuals died. There was no significant difference between the discriminative ability of BODE_6MWT_ (C-index 0.709, 95% confidence interval [CI], 0.680–0.737), BODE_SPPB_ (C-index 0.683, 95% CI, 0.647–0.712), BODE_4MGS_ (C-index 0.676, 95% CI, 0.643–0.700) and BODE_BALANCE_ (C-index 0.686, 95% CI, 0.651–0.713) for predicting mortality.

**Conclusions:**

the SPPB, and its 4MGS and balance components, can potentially be used as an alternative to the 6MWT in the BODE Index without significant loss of predictive ability in all-cause mortality.

## Key points

- COPD is common amongst older individuals, accurate prediction of mortality risk improves care planning.
- The BODE Index, a mortality index for COPD, has failed to be widely adopted, likely due to the impractical 6MWT component.
- SPPB, and its 4MGS and balance components, is a practical alternative to 6MWT without significant loss in predicting mortality.

## Introduction

Chronic obstructive pulmonary disease (COPD) is predominantly a disease of older people, with a prevalence of ~5% in individuals aged <65 years but rising to 20–25% in those aged >85 [1]. Importantly, like other diseases prevalent in older people, COPD is recognised to involve multiple organ systems with recognised extrapulmonary manifestations such as frailty, cardiovascular disease, osteoporosis and cognitive changes [2,3], as well as changes in skeletal muscle, which appear directly related to COPD [4]. COPD has also been considered on the basis of telomere shortening to be a disease of premature ageing [5].

Prognostication for people living with COPD is key for future care planning. Accurately predicting prognosis also helps people better understand their condition and decide on safety or desirability of interventional procedures such as general anaesthesia or chemotherapy. Mortality prediction is equally important for clinical guidelines and government health departments to plan for healthcare system delivery and funding provision.

The best known prognostic index for COPD is the BODE Index, a composite score of body mass index (BMI), spirometry, breathlessness and exercise capacity defined by the 6-minute walk test (6MWT), which on its own is predictive of all-cause mortality [6,7]. However, both BODE and 6MWT have received limited adoption in clinical practise globally, likely because the 6MWT requires a 30-m corridor and a training walk [8]; thus, the 6MWT in practise takes >30 minutes. Unsurprisingly, the NICE (National Institute for Health and Care Excellence) UK 2018 guidelines for COPD advised against the BODE Index for prognostication in COPD. Since the other three components of the BODE Index can be measured with relative ease, it is likely that the 6MWT is an important barrier to more widespread use of the BODE Index in clinical settings, and yet assessment of functional capacity contributes significantly to mortality prediction.

Several studies have tried to improve the BODE Index by exploring additional markers; replacing the 6MWT component with alternative measures of exercise capacity such as the incremental shuttle walk test [9] and maximum oxygen uptake [10] has shown equivalence. However, both tests are at least as time consuming and resource intensive as the 6MWT. Celli *et al.* [11] demonstrated that the addition of interleukin 6 to the BODE Index only improved the models’ predictive performance marginally, and venepuncture is again resource intensive. Interestingly, adding exacerbation history has not been shown to substantially improve the prognostic capacity of the BODE Index [12].

Recognition of a lack of suitable predictive biomarkers has led to extensive research to evaluate functional assessments that can be easily used in the clinical setting. Of the many different assessments studied, the short physical performance battery (SPPB), a composite score of 4-m gait speed test (4MGS), balance and chair stand, is an attractive tool that fits these criteria. It has been validated in different clinical settings, is prognostic for mortality in older individuals in the general population and takes <5 minutes to complete [13–15].

We hypothesised that the SPPB may have comparable predictive power to the 6MWT, and could thus replace it within the BODE Index. We firstly aimed to evaluate the association between all-cause mortality with 6MWT, SPPB and its components in COPD patients. Next, we sought to investigate whether a BODE Index in which the 6MWT was replaced by either the SPPB or its components retained predictive ability. Finally, we aimed to assess whether addition of the SPPB or its individual components to the BODE Index improved the predictive ability for all-cause mortality.

## Methods

### Study design and participants

The Evaluation of the Role of Inflammation in Chronic Airways disease (ERICA) study is a multicentre observational study of 729 stable global initiative for obstructive lung disease (GOLD) stage II–IV COPD patients, registered with the UK Clinical Trials Gateway. Co-morbidities were not considered as exclusion criteria. Full details of the protocol and baseline results are provided elsewhere [16] (Supplementary Text S1). Following baseline assessments, mortality data were obtained from the UK Office for National Statistics (ONS). Analyses presented here were limited to a maximum of 3 years of follow-up that ended in August 2016.

Informed written patient consent was obtained from all study participants. The National Research Ethics Service Committee East of England—Cambridge South (reference 11/EE/0357) approved the study.

### Point assignment for components of BODE Index and SPPB

The BODE Index is a multidimensional weighted model. The model is made up of BMI (kg/m^2^), Obstruction (FEV_1_% predicted), Dyspnoea (Medical Research Council [MRC] score) and Exercise capacity (6MWT distance) [6].

Short physical performance battery is a lower-extremity physical assessment consisting of three separate components scored 0–4 each, comprising the 4MGS, standing balance and five repetition chair stand test [17,18]. The only equipment required is a stopwatch, standard chair and measuring tape to mark a distance of 4 m. Total SPPB score ranges 0–12 points, with higher scores indicating better performance. To preserve a four-category system, we combined one and two points.

Points for BODE Index (0–10) were assigned in quartiles (Supplementary Table S2), with higher scores indicating higher risk of mortality [6]. The SPPB itself was divided into 10–12, 7–9, 4–6 and <4 based on the distribution of data and cut-off score of <10 to define functional limitation [19,20].

### Statistical analysis

Hazard ratios (HR) with 95% confidence intervals (CI) were estimated using multivariable Cox regression. All statistical models were stratified by recruitment centre, adjusted for age and sex and developed according to the TRIPOD and guidelines for clinical prediction models. The preselected prediction models were: (i) BMI, (ii) BMI + MRC dyspnoea, (iii) BMI + MRC dyspnoea + FEV_1_%, (iv) BMI + MRC dyspnoea + FEV_1_% + 6MWT, (v) BMI + MRC dyspnoea + FEV_1_% + SPPB. Linearity of continuous predictors was assessed visually. We tested for violation of the proportional hazards assumption by including time interactions and visually examining Arjas plots. Discrimination (i.e. Harrell’s C-statistic [21,22]) and calibration (i.e. Gronnesby and Borgan test [23] and calibration plots) were assessed using 10-fold cross-validation with 200 replications [24]. Effect of missing data was assessed in sensitivity analyses using multivariable imputation by chained equations (MICE). Detailed methods are provided in the online supplement (Supplementary Text S2).

Analyses were performed using Stata version 13.0 (College Station, TX) and R (R Foundation). Observational data are reported according to the STROBE statement. All tests were two-sided and statistical significance was defined by 95% CI for HRs not traversing 1 or *P* value <0.05.

## Results

Of 714 individuals with COPD followed by the ONS for survival status, 630 had complete baseline data and were included in the primary analysis (Supplementary Figure S1); of these 630 individuals, 386 (61%) were male, 192 (30%) current smokers, 358 (57%) GOLD grade II and the median age was 67 years (range 43–84 years; Table 1 and Supplementary Tables S3–S5). In total, 245 (39%) had functional limitation (SPPB score <10) with a median (interquartile range [IQR]) SPPB score of 10 (8–12) and 6MWT distance of 370 (268–440) m.

**Table 1:** Baseline characteristics (analytical population, *n* = 630).

Characteristics	All individuals (%)
**Description**	
Age (years), median (IQR)	67 (62–73)
Male	386 (61)
BMI (kg/m^2^), median (IQR)	27 (23–31)
**Lung function**	
FEV_1_% predicted, median (IQR)	53 (40–65)
Current smoker	192 (30)
MRC dyspnoea score	
1	54 (9)
2	261 (41)
3	138 (22)
4	125 (20)
5	52 (8)
GOLD	
Stage II	358 (57)
Stage III	216 (34)
Stage IV	56 (9)
**Musculoskeletal measures**	
6MWT distance (m), median (IQR)	370 (268–440)
SPPB (0–12), median (IQR)	10 (8–12)
No functional limitation ≥10	385 (61)
Functional limitation <10	245 (39)
4MGS score (0–4), median (IQR)	4 (3–4)
Balance points (0–4), median (IQR)	4 (4–4)
Chair stand score (0–4), median (IQR)	3 (1–4)
QMVC peak (kg), median (IQR)	30 (22–39)

Values are given as the median and IQR, or no. of cases (%). Baseline data of 630 individuals are included. QMVC, quadriceps maximum voluntary contraction.

### Factors associated with all-cause mortality

Sixty patients (10%) died during the follow-up period. The three-year survival probability was 90% (88–93% CI) with an event rate of 3.3 (95% CI, 2.6–4.3) per 100 person-years. Age-adjusted multivariable analysis identified multiple markers associated with mortality including BMI (HR 0.91 per 1-point increase, 95% CI, 0.86–0.97, *P* = 0.002), 6MWT (HR 0.85 per 30-m increase, 95% CI, 0.78–0.92, *P* < 0.001), SPPB (HR 0.81 per 1-point increase, 95% CI, 0.72–0.92, *P* = 0.002), 4MGS (HR 0.67 per 1-point increase, 95% CI, 0.49–0.93, *P* = 0.015) and balance (HR 0.63 per 1-point increase, 95% CI, 0.48–0.82, *P* = 0.001), Supplementary Figure S2 and Supplementary Table S6). Chair stand was not associated with all-cause mortality in multivariable analysis.

**Figure 1:**
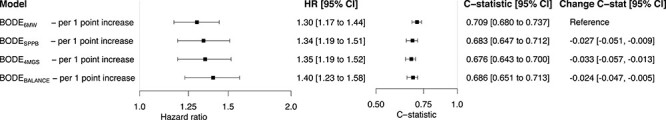
HRs and C-indices with change scores for various BODE models. All models were stratified by recruitment centre.

### Predictive models

**Figure 2:**
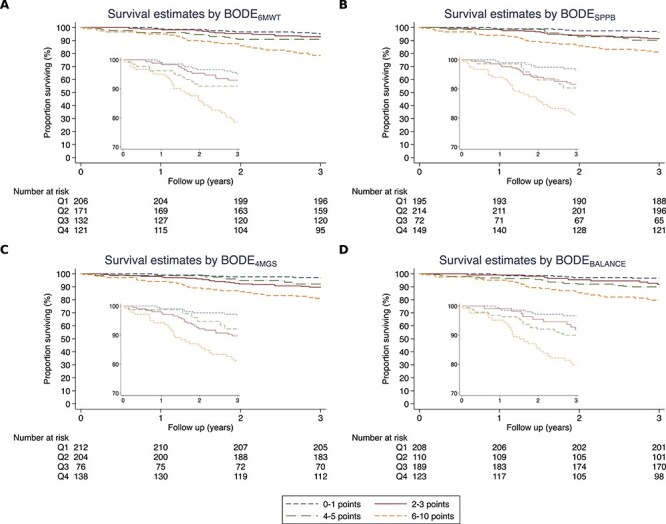
Survival risk indices, by quartiles. (A) BODE_6MWT_, (B) BODE_SPPB_, (C) BODE_4MGS_ and (D) BODE_BALANCE_. Mortality data obtained from the UK ONS.

Predictive modelling indicated slightly higher HR for SPPB and its components compared with BODE_6MWT_ (Figure 1 and Supplementary Figure S3). The C-statistic was the highest for BODE_6MWT_ (C = 0.709, 95% CI, 0.680–0.737), but there was no significant difference in discriminative ability compared with BODE_SPPB_ (C = 0.683, 95% CI, 0.647–0.712; Figure 1 and Table 2). Neither was there a significant difference in risk discrimination when compared with the BODE_4MGS_ (C = 0.676, 95% CI, 0.643–0.700) or BODE_BALANCE_ (C = 0.686, 95% CI, 0.651–0.713). When comparing BODE_SPPB_ with its components, there were no significant differences in risk discrimination between indices. Calibration tests and plots of the hazard models indicate good model fit and calibration for 3-year prediction of mortality (Table 2 and Supplementary Figure S4, and Supplementary Text S3). Risk quartile estimates for each BODE model are displayed using Kaplan–Meier plots (Figure 2).

**Table 2:** Cox proportional hazards regression analyses for all-cause mortality, using continuous data.

Model	Model 1: BMI	Model 2: BMI, MRC	Model 3: BMI, MRC, FEV_1_%	Model 4: BODE_6MWT_	Model 5: BODE_SPPB_	Model 6: BODE_4MGS_	Model 7: BODE_BALANCE_
Variables	HR (95% CI)						
BMI—per 1-point increase	0.90 (0.85–0.95)	0.91 (0.86–0.95)	0.91 (0.87–0.96)	0.89 (0.85–0.94)	0.90 (0.86–0.95)	0.91 (0.86–0.95)	0.91 (0.87–0.96)
MRC dyspnoea score—per 1-point increase		1.38 (1.10–1.72)	1.27 (0.99–1.63)	0.92 (0.68–1.23)	1.05 (0.80–1.38)	1.15 (0.89–1.49)	1.15 (0.90–1.49)
FEV_1_—per 5% increase % predicted			0.93 (0.85–1.02)	0.98 (0.89–1.07)	0.93 (0.84–1.02)	0.94 (0.85–1.03)	0.92 (0.83–1.01)
6MWT—per 30-m increase				0.84 (0.78–0.91)			
SPPB (component)—per increase of 1 point					0.80 (0.71–0.90)	0.61 (0.45–0.84)	0.63 (0.49–0.82)
C-index	0.634 (0.600–0.658)	0.650 (0.620–0.676)	0.646 (0.607–0.672)	0.709 (0.680–0.737)	0.682 (0.647–0.712)	0.676 (0.642–0.700)	0.685 (0.651–0.712)
Goodness of fit, chi2(3)	10.63	2.57	1.95	2.64	1.57	0.89	4.28
*P* > chi2	0.014	0.464	0.583	0.451	0.665	0.827	0.233
Change in C-statistic	−0.075 (−0.106 to −0.048)	−0.060 (−0.082 to −0.037)	−0.064 (−0.083 to −0.041)	Reference	−0.027 (−0.052 to −0.009)	−0.033 (−0.057 to −0.013)	−0.024 (−0.047 to −0.005)

All models were stratified by recruitment centre. Goodness of fit estimates was based on quartiles of risk. FEV_1_% = predicted forced expiratory volume 1 second.

Compared with the composite scoring, use of continuous data did not significantly improve discriminative ability for any of the BODE indices (Supplementary Table S7). When assessing BODEs’ individual scoring components, most of BODEs’ predictive ability was attributed to 6MWT (C = 0.648, 95% CI, 0.609–0.673; Supplementary Figure S5). When replacing 6MWT with the SPPB, or its components 4MGS or balance, the C-index changed from 0.671 (95% CI, 0.641–0.693) to 0.667 (95% CI, 627–0.694), 0.670 (95% CI, 0.634–0.694) and 0.682 (95% CI, 0.646–0.702), respectively. The C-index (95% CI) for the SPPB total score alone was 0.617 (0.580–0.645), 0.602 (0.536–0.639) for balance and 0.600 (0.545–0.631) for 4MGS.

## Sensitivity analysis

All 714 individuals (*n* = 71 deaths after 3 years of follow-up) were included in sensitivity analyses using multiple imputation of missing baseline values (Supplementary Figures S6–S9). Cross-validated C-indices decreased but were unchanged between the different models (Supplementary Table S8).

## Discussion

Short physical performance battery or its 4MGS and balance components can replace 6MWT in BODE for the prediction of all-cause mortality in stable COPD patients without loss of predictive power. The study confirms prior observations that the total SPPB or 4MGS or balance test individually is associated with prognosis in simple age- and sex-adjusted analysis.

Older patients are frequently co-morbid and have functional limitation. It is this property that is reflected in the BODE score. Often a patient’s consideration for therapies, for example surgery or chemotherapy, with significant side effects or which are resource intensive may be determined by their overall prognosis. In this context, a diagnosis of COPD may be used as a reason not to offer therapies that are otherwise beneficial. Thus, being able to determine an accurate medium-term prognosis is often helpful in determining the best advice for patients and the most appropriate use of resources. In the present study, the average age of participants was 67 with an IQR 62–73 making them very typical of older patients with COPD in the UK.

The majority of our cohort could be classified as ‘normal’ physical function based on the SPPB and very few patients had low SPPB scores. Furthermore, the majority of our patients had low BODE Index scores. This is important to consider when interpreting our results, and is likely reflective of a general outpatient population of patients with COPD rather than a more severe COPD population engaging in pulmonary rehabilitation, therefore making the results from our models more generalisable to clinicians seeing patients in a primary or secondary outpatient setting. The study population in which a model is examined is important, especially when considering that most models are not externally validated due to limitations in similarities across study populations [25].

Unlike 6MWT, the SPPB is simple to measure, requiring only a standard chair, stopwatch (or smartphone) and a 4-m flat surface, taking <5 minutes. In 2018, the European Medicines Agency approved the SPPB as a measure of frailty for diseases associated with musculoskeletal decline [26]. Furthermore, our data suggest that even substitution of a single component (e.g. balance) does not result in any significant loss in predictive ability compared with BODE_6MWT_.

We were unable to demonstrate any significant improvement in the predictive ability of the BODE_6MWT_ by adding the SPPB as an additional test. Strong correlations between 6MWT and SPPB (and its components) have been described previously [27] and were to be expected as they both assess lower limb function (Supplementary Figure S10). We suspect this explains not only why the SPPB can be easily substituted for 6MWT but also why it conferred no additional value when added to 6MWT.

The superiority of the balance component is of interest and may reflect that balance is an integrative test reflecting multiple pathologies or the effects of polypharmacy [28] beyond musculoskeletal weakness. Since, only 128 (20%) of our cohort had a score below the maximum four points, this may indicate that there is a threshold effect where any reduction in the balance score confers a higher risk of mortality.

This study has limitations. Firstly, there is no independent validation cohort with a fully comparable data set. We mitigated this issue using a cross-validation technique approach and estimated C-indices through random partitioning of the dataset. Secondly, baseline data differed amongst the recruitment centres but was addressed through stratification by centre. Thirdly, there were missing data with evidence that some were not at random (Supplementary Figure S11). Analysing complete-case data may have introduced bias, and although HRs and C-statistics of the models shifted following MICE, the main conclusions were unchanged.

Additional deaths occurred beyond the 3 years of follow-up included in the primary analysis. However, 3 years of follow-up was chosen because insufficient deaths occur over a shorter time frame, whereas over a long time period, the predictive ability of BODE diminishes, both because ageing is a strong predictive variable and because measured variables at baseline are so distant from the point of death. Consistent with this, some very large COPD trials such as the TOwards a Revolution in COPD Health (TORCH) [29] and Study to Understand Mortality and Morbidity in COPD (SUMMIT) [30] have used 3-year follow-up, and furthermore, this is the time frame that regulatory agencies such as the Food and Drug Administration and European Medicines Agency also consider for outcomes data in COPD. Pragmatically when considering the wisdom of an unrelated intervention in an older person, 3 years is a reasonable time frame.

## Conclusion

The SPPB, and its 4MGS and balance components, can potentially be used as an alternative to the 6MWT in the BODE Index without significant loss of predictive ability in all-cause mortality. Adoption of the SPPB might potentially enhance the uptake of risk indices such as the BODE Index, and subsequently prognostication of COPD, in clinical practise.

## Supplementary Material

Supplementary_update_afaa138
