# Supplementary material for: Short physical performance battery as a practical tool to assess mortality risk in chronic obstructive pulmonary disease
Source: Age Ageing. 2020 Sep 7;50(3):795–801. doi: 10.1093/ageing/afaa138 (PMC8098797; doi:10.1093/ageing/afaa138)
Supplement: Supplementary_update_afaa138 [file supplementary_update_afaa138.docx]

**Short physical performance battery as a practical tool to assess mortality risk in chronic obstructive pulmonary disease.**

Table of Contents

[Supplementary Text 3](#_Toc44943756)

[**Text S1.** Evaluating the role of inflammation in chronic airways disease (ERICA) Methods. 3](#_Toc44943757)

[**Text S2.** Details of multiple imputation by chained equations. 3](#_Toc44943758)

[**Text S3.** Practical example of using the BODE Index. 3](#_Toc44943759)

[Supplementary Tables 5](#_Toc44943760)

[**Table S1.** Multidimensional risk factors, published prediction models for the prediction of mortality in COPD. 5](#_Toc44943761)

[**Table S2.** Assignment of points for BODE and SPPB. 7](#_Toc44943762)

[**Table S3.** Self-reported baseline characteristics. 7](#_Toc44943763)

[**Table S4.** Self-reported baseline characteristics, by recruitment centre. 9](#_Toc44943764)

[**Table S5.** Baseline characteristics by BODE_SPPB_ quartiles. 10](#_Toc44943765)

[**Table S6.** Adjusted multivariable associations, with the occurrence of death, by years of follow-up. 11](#_Toc44943766)

[**Table S7.** Cox proportional hazards regression analyses for all-cause mortality during follow-up, using composite scores for BODE indices.^a^ 13](#_Toc44943767)

[**Table S8.** Cox proportional hazards regression analyses for all-cause mortality, using continuous data after multiple imputation. 14](#_Toc44943768)

[**Table S9.** Risk indices using point system, by survival status. 15](#_Toc44943769)

[**Table S10.** Risk indices using point system, by cause of death. 15](#_Toc44943770)

[Supplementary Figures 16](#_Toc44943771)

[**Figure S1.** Participant enrolment flow diagram with three years follow up of mortality. 17](#_Toc44943772)

[**Figure S2.** Forest plot displaying standardized adjusted hazard ratios, by years of follow-up. 19](#_Toc44943773)

[**Figure S3.** Change in C-index of BODE with additional components. 20](#_Toc44943774)

[**Figure S4.** Predicted and observed mortality by risk quartiles with associated 95% confidence intervals. 20](#_Toc44943775)

[**Figure S5.** Change scores C-index. Individual components BODE. 21](#_Toc44943776)

[**Figure S6.** Missing data patterns. 22](#_Toc44943777)

[**Figure S7.** Boxplots, after multiple imputation. 23](#_Toc44943778)

[**Figure S8.** Kernel density plots, after multiple imputation. 23](#_Toc44943779)

[**Figure S9.** Diagnostic histograms, after multiple imputation. Examples of first imputed dataset only. 24](#_Toc44943780)

[**Figure S10.** Correlation matrix: Associations among the variables measured at baseline. 25](#_Toc44943781)

[**Figure S11.** Missing data patterns. 26](#_Toc44943782)

## Supplementary Text

### **Text S1.** Evaluating the role of inflammation in chronic airways disease (ERICA) Methods.

The ERICA study is a multi-centre observational, non-interventional, epidemiological cohort study, with a sample size of 729 COPD patients, established to identify important cardiovascular and musculoskeletal biomarkers that could be targeted to improve the outcomes of COPD patients. The ERICA dataset is a data collection tied to capture events and changes related to respiratory, musculoskeletal and cardiovascular function including changes in therapy allowing to investigate the prevalence and significance of cardiovascular and musculoskeletal manifestations of COPD. Five UK centres with an interest in COPD undertook this study: Cambridge (n = 88), Edinburgh (n = 100), Cardiff (n = 374), Nottingham (n = 106) and London (n = 61). The ERICA study is part of a consortium based on a partnership between academia and industry. The consortium includes additional cohort studies such as ECLIPSE and ARCADE.^[[1]](#footnote-1),^^[[2]](#footnote-2)^ The patient population includes adults aged ≥ 40 years with a clinical diagnosis of COPD, post-bronchodilator spirometry FEV_1_/FVC ratio < 0.7 and FEV_1_ ≤ 80% of predicted normal, current or ex-smoker with a smoking history of at least ten pack years, and who were clinically stable for more than four weeks from any exacerbation requiring treatment with oral steroids or antibiotics or hospitalisation were eligible.^[[3]](#footnote-3)^ Patients were recruited between October 2010 and February 2011 in the study participating centres. Mortality data was obtained from the UK Office for National Statistics last updated in November 2017. Ethics approval and written informed consent has been obtained. Body mass index was categorised according to the levels defined by the World Health Organization (WHO).^[[4]](#footnote-4)^ Global initiative for obstructive lung disease (GOLD) stages were estimated as described by the GOLD.^[[5]](#footnote-5)^ Functional limitation was defined by a SPPB cut-off score of < 10.^[[6]](#footnote-6),^^[[7]](#footnote-7)^

### **Text S2.** Details of multiple imputation by chained equations.

Predictive mean matching was used for continuous variables, ordered logistic regression (as continuous) for ordinal variables, multinomial logistic regression for categorical variables, and logistic regression for binary variables. Derived variables such as SPPB (a composite score of 4MGS, balance, and chair stand) were estimated post MICE using passive imputation. To minimise potential overfitting caused from using the same imputed dataset for the training and test data for 10-fold cross-validation, we created 10 x 2 imputed datasets and used ten for model derivation and the other ten for model validation. Thus, within each step of the cross-validation, the training and test datasets were from two different imputations. We performed cross-validation separately for the ten pairs of imputed datasets before combining estimates of interest using Rubin’s rules. Comparison of confidence intervals between models allowed assessment of significant differences, with overlap of confidence intervals denoting no significant difference in discriminative ability.

### **Text S3.** Practical example of using the BODE Index.

When using the BODE_SPPB_, an individual with a BMI of ≤ 21 (1 point), FEV_1_% predicted of 36-49% (2 points), dyspnoea score of 4 (3 points), and SPPB score of 4-6 (2 points), has a total score of eight points out of ten. The BODE_SPPB_ Index quartile 1 was defined by a score of 0-1, quartile 2 by a score of 2-3, quartile 3 by a score of 4, and quartile 4 by a score of 5-10. A BODE_SPPB_ Index score of eight would then indicate a 24% predicted risk of mortality over the next three years. Specifically, of 100 individuals with the same BODE_SPPB_ score, 24 would die within the next three years.

## Supplementary Tables

### **Table S1.** Multidimensional risk factors, published prediction models for the prediction of mortality in COPD.

Placed in order of publication date.

|  | BODE (0-10), Celli *et al.* 2004 | HADO (0-12), Esteban *et al.* 2006 | mBODE (0-10), Cote *et al.* 2007 | CPI (0-100), Briggs *et al.* 2008 | DOREMI BOX (0-10), Kostianev *et al.* 2008 | ADO (0-10), Puhan *et al.* 2009 | U-BODE (0-11), Puhan *et al.* 2009 | BODEx (0-9), Soler-Cataluna *et al.* 2009 | eBODE (0-12), Soler-Cataluna *et al.* 2009 | PILE (0-10), Mehrotra *et al.* 2010 | ECLIPSE, Celli *et al.* 2012 | BODE-A, Stolz *et al.* 2014 |
| --- | --- | --- | --- | --- | --- | --- | --- | --- | --- | --- | --- | --- |
| Age |  |  |  |  |  |  |  |  |  |  |  |  |
| Sex |  |  |  |  |  |  |  |  |  |  |  |  |
| BMI |  |  |  |  |  |  |  |  |  |  |  |  |
| Dyspnoea (MRC, ATS, Fletcher, CRQ) |  |  |  |  |  |  |  |  |  |  |  |  |
| FEV_1_ % |  |  |  |  |  |  |  |  |  |  |  |  |
| 6MWT distance |  |  |  |  |  |  |  |  |  |  |  |  |
| Exacerbation |  |  |  |  |  |  |  |  |  |  |  |  |
| Exercise max. O_2_ consumption |  |  |  |  |  |  |  |  |  |  |  |  |
| O_2_-use |  |  |  |  |  |  |  |  |  |  |  |  |
| CVD |  |  |  |  |  |  |  |  |  |  |  |  |
| Blood Oxygen (PaO_2_) |  |  |  |  |  |  |  |  |  |  |  |  |
| Health status |  |  |  |  |  |  |  |  |  |  |  |  |
| Activity |  |  |  |  |  |  |  |  |  |  |  |  |
| QMVC |  |  |  |  |  |  |  |  |  |  |  |  |
| Inflammatory markers (e.g. IL-6 and fibrinogen) |  |  |  |  |  |  |  |  |  |  |  |  |
| Total sample size | 625 | 611 | 444 | 8802 | 68 | 232 | 232 | 185 | 185 | 268 | 1843 | 549 |
| Observed deaths (total No.) | 162 | 94 | 206 | 166 | 22 | 79 | 79 | 71 | 71 | 83 | 168 | 26 |
| Follow-up (months) | 28 | 36 | 22 | 6-36 | 36 | 30 | 30 | 36 | 36 | 73 | 36 | 12 |
| C-statistic | 0.74 ^[[8]](#footnote-8)^ | 0.68 ^[[9]](#footnote-9)^ | 0.72 ^[[10]](#footnote-10)^ | 0.71 ^[[11]](#footnote-11)^ | NA ^[[12]](#footnote-12)^ | 0.63 ^[[13]](#footnote-13)^ | 0.61 ^13^ | 0.74 ^[[14]](#footnote-14)^ | 0.77 ^14^ | 0.71 ^[[15]](#footnote-15)^ | 0.73 ^[[16]](#footnote-16)^ | 0.75 ^[[17]](#footnote-17)^ |

ATS = American Thoracic Society. BMI = Body Mass Index. CRQ = Chronic Respiratory Questionnaire. CVD = Cardiovascular Disease. FEV_1_ = forced expiratory volume in one second. IL6 = Interleukin 6. MRC = Medical Research Council. O_2_ = Oxygen. PaO_2_ = Partial oxygen pressure. QMVC = quadriceps maximum voluntary contraction. 6MWT = six-minute walk test.

### **Table S2.** Assignment of points for BODE and SPPB.

| **Variable** | **0 points** | **1 point** | **2 points** | **3 points** |
| --- | --- | --- | --- | --- |
| BODE |  |  |  |  |
| BMI (kg/m^2)^ | > 21 | ≤ 21 |  |  |
| FEV_1_ (% predicted) | ≥ 65 | 50-64 | 36-49 | ≤ 35 |
| Dyspnoea (MRC scale) | 0-1 | 2 | 3 | 4 |
| Six-minute walk test distance (m) | ≥ 350 | 250-349 | 150-249 | ≤ 149 |
| **Alternative musculoskeletal measures** |  |  |  |  |
| SPPB (points) | 10-12 | 7-9 | 4-6 | < 4 |
| Four-metre gait speed (points) | 4 | 3 | 1-2 | 0 |
| Balance (points) | 4 | 3 | 1-2 | 0 |
| Chair stand (points) | 4 | 3 | 1-2 | 0 |

BMI = body-mass index. FEV_1_ = forced expiratory volume in one second. MRC = Medical Research Council. SPPB = short physical performance battery.

### **Table S3.** Self-reported baseline characteristics.

| Characteristic | **Total (%)** | **SPPB, ≤ 9 points** | **SPPB ≥ 10 points** | ***P* value** |
| --- | --- | --- | --- | --- |
| **Description** |  |  |  |  |
| Age (yrs.), median (IQR) | 67 (62-73) | 70 (63-75) | 66 (62-71) | < 0.001 |
| Sex  Male  Female | 386 (61)  244 (39) | 129 (53)  116 (47) | 257 (67)  128 (33) | < 0.001 |
| Body mass index (kg/m^2^), median (IQR) | 27 (23-31) | 28 (24-32) | 26 (23-29) | < 0.001 |
| **Lung function** |  |  |  |  |
| FEV_1_ %predicted, median (IQR) | 53 (40-65) | 52 (39-63) | 54 (41-66) | 0.265 |
| Smoking status  Current  Former | 192 (30)  438 (70) | 73 (30)  172 (70) | 119 (31)  266 (69) | 0.767 |
| MRC dyspnoea score  1  ≥ 2 | 54 (9)  576 (91) | 7 (3)  238 (97) | 47 (12)  338 (88) | < 0.001 |
| GOLD, n (%)  Stage II  Stage III  Stage IV | 358 (57)  216 (34)  56 (9) | 131 (53)  89 (36)  25 (10) | 227 (59)  127 (33)  31 (8) | 0.357 |
| **Musculoskeletal measures** |  |  |  |  |
| 6MWT distance (metre), median (IQR) | 370 (268-440) | 265 (174-344) | 420 (360-470) | < 0.001 |
| 4MGS score (0-4), median (IQR) | 4 (3-4) | 3 (3-4) | 4 (4-4) | < 0.001 |
| Balance points (0-4), median (IQR) | 4 (4-4) | 4 (3-4) | 4 (4-4) | < 0.001 |
| Chair stand score (0-4), median (IQR) | 3 (1-4) | 1 (1-1) | 3 (3-4) | < 0.001 |
| QMVC peak (kg), median (IQR) | 30 (22-39) | 25 (19-33) | 32 (26-41) | < 0.001 |

Values are given as the median and interquartile range (IQR), or No. of cases (%). Baseline data of 630 patients are included. P-values estimated using Wilcoxon-Mann-Whitney test for continuous data, and Chi-square test for categorical data.

FEV_1_ = forced expiratory volume in one second. GOLD = global initiative for obstructive lung disease. MRC = Medical Research Council. QMVC = quadriceps maximum voluntary contraction. SPPB = short physical performance battery. 4MGS = four-metre gait speed. 6MWT = six-minute walk test.

### **Table S4.** Self-reported baseline characteristics, by recruitment centre.

| Characteristic | **Total (n = 630)** | **Cambridge (n = 73)** | **Edinburgh (n = 89)** | **Cardiff (n = 328)** | **Nottingham (n = 84)** | **London (n = 59)** | ***P* value** |
| --- | --- | --- | --- | --- | --- | --- | --- |
| **Description** |  |  |  |  |  |  |  |
| Age (yrs.), median (IQR) | 67 (62-73) | 69 (64-74) | 69 (64-74) | 67 62-73) | 68 (62-72) | 65 (60-72) | 0.129 |
| Sex  Male  Female | 386 (61)  244 (39) | 59 (81)  14 (19) | 52 (58)  37 (42) | 179 (55)  149 (45) | 58 (69)  26 (31) | 38 (68)  18 (32) | < 0.001 |
| Body mass index (kg/m^2^), median (IQR) | 27 (23-31) | 26 (23-30) | 25 (22-29) | 28 (24-32) | 26 (22-31) | 23 (20-26) | < 0.001 |
| **Lung function** |  |  |  |  |  |  |  |
| FEV_1_% predicted, median (IQR) | 53 (40-65) | 51 (35-60) | 52 (40-66) | 55 (43-67) | 53 (42-63) | 41 (27-60) | < 0.001 |
| Smoking status  Current  Former | 192 (30)  438 (70) | 12 (16)  61 (84) | 30 (34)  59 (66) | 122 (37)  206 (63) | 21 (25)  63 (75) | 7 (13)  49 (88) | < 0.001 |
| MRC dyspnoea score  1  ≥ 2 | 54 (9)  576 (91) | 10 (14)  63 (86) | 5 (6)  84 (94) | 21 (6)  307 (94) | 14 (17)  70 (83) | 4 (7)  52 (93) | 0.019 |
| GOLD, n (%)  Stage II  Stage III  Stage IV | 358 (57)  216 (34)  56 (9) | 39 (53)  23 (32)  11 (15) | 49 (55)  37 (42)  3 (3) | 203 (62)  106 (32)  19 (6) | 47 (56)  33 (39)  4 (5) | 20 (36)  17 (30)  19 (34) | < 0.001 |
| **Musculoskeletal measures** |  |  |  |  |  |  |  |
| 6MWT distance (metre), median (IQR) | 370 (268-440) | 420 (286-500) | 407 (326-480) | 353 (240-420) | 355 (255-419) | 431 (304-482) | < 0.001 |
| SPPB (0-12), median (IQR | 10 (8-12) | 11 (10-12) | 11 (9-12) | 10 (7-11) | 11 (9-12) | 11 (10-12) | < 0.001 |
| 4MGS score (0-4), median (IQR) | 4 (3-4) | 4 (4-4) | 4 (4-4) | 4 (3-4) | 4 (4-4) | 4 (4-4) | < 0.001 |
| Balance points (0-4), median (IQR) | 4 (4-4) | 4 (4-4) | 4 (4-4) | 4 (3-4) | 4 (4-4) | 4 (4-4) | < 0.001 |
| Chair stand score (0-4), median (IQR) | 3 (1-4) | 3 (3-4) | 3 (2-4) | 2 (1-3) | 3 (1-4) | 3 (3-4) | < 0.001 |
| QMVC peak (kg), median (IQR) | 30 (22-39) | 31 (26-42) | 30 (24-38) | 29 (20-38) | 31 (25-42) | 31 (23-36) | 0. 006 |
| Event rate, per 100 person-years (95% CI) | 3.3 (2.6 to 4.3) | 2.4 (1.0 to 5.8) | 3.6 (1.9 to 7.0) | 2.8 (1.9 to 4.1) | 3.3 (1.7 to 6.6) | 7.1 (3.9 to 12.8) | ~ |

Values are given as the median and interquartile range (IQR), or No. of cases (%). Baseline data of 630 patients are included. P-values estimated using Analysis of variance (ANOVA) for continuous data, and Chi-square test or Fisher exact test for categorical data.

FEV_1_ = forced expiratory volume in one second. GOLD = global initiative for obstructive lung disease. MRC = Medical Research Council. QMVC = quadriceps maximum voluntary contraction. SPPB = short physical performance battery. 4MGS = four-metre gait speed. 6MWT = six-minute walk test.

### **Table S5.** Baseline characteristics by BODE_SPPB_ quartiles.

| Characteristic | All individuals (%) | BODE_SPPB_Q1_ | BODE_SPPB_Q2_ | BODE_SPPB_Q3_ | BODE_SPPB_Q4_ | *P* |
| --- | --- | --- | --- | --- | --- | --- |
| **Description** |  |  |  |  |  |  |
| Age (yrs.), median (IQR) | 67 (62-73) | 67 (63-72) | 68 (62-73) | 67 (61-73) | 67 (62-73) | 0.881 |
| Male | 386 (61) | 132 (68) | 123 (57) | 46 (64) | 85 (57) | 0.111 |
| Body mass index (kg/m^2^), median (IQR) | 27 (23-31) | 27 (25-31) | 26 (23-30) | 27 (23-31) | 25 (20-32) | 0.006 |
| **Lung function** |  |  |  |  |  |  |
| FEV_1_ %predicted, median (IQR) | 53 (40-65) | 67 (59-74) | 52 (42-62) | 46 (37-51) | 35 (27-45) | <0.001 |
| Current smoker | 192 (30) | 55 (28) | 71 (33) | 19 (26) | 47 (32) | 0.602 |
| MRC dyspnoea score  1  2  3  4  5 | 54 (9)  261 (41)  138 (22)  125 (20)  52 (8) | 37 (19)  146 (75)  12 (6)  0 (0)  0 (0) | 17 (8)  104 (49)  64 (30)  28 (13)  1 (0) | 0 (0)  10 (14)  31 (43)  29 (40)  2 (3) | 0 (0)  1 (1)  31 (21)  68 (46)  49 (33) | <0.001 |
| GOLD  Stage II  Stage III  Stage IV | 358 (57)  216 (34)  56 (9) | 195 (100)  0 (0)  0 (0) | 117 (55)  94 (44)  3 (1) | 22 (31)  43 (60)  7 (10) | 24 (16)  79 (53)  46 (31) | <0.001 |
| **Musculoskeletal measures** |  |  |  |  |  |  |
| 6MWT distance (metre), median (IQR) | 370 (268-440) | 439 (383-490) | 383 (305-438) | 310 (222-384) | 240 (155-318) | <0.001 |
| SPPB (0-12), median (IQR)  No functional limitation, ≥10  Functional limitation < 10  4MGS score (0-4), median (IQR)  Balance points (0-4), median (IQR)  Chair stand score (0-4), median (IQR) | 10 (8-12)  385 (61)  245 (39)  4 (3-4)  4 (4-4)  3 (1-4) | 11 (10-12)  174 (89)  21 (11)  4 (4-4)  4 (4-4)  3 (3-4) | 11 (9-12)  132 (62)  82 (38)  4 (3-4)  4 (4-4)  3 (1-4) | 10 (8-11)  36 (50)  36 (50)  4 (3-4)  4 (4-4)  2 (1-3) | 8 (6-10)  43 (29)  106 (71)  3 (3-4)  4 (3-4)  1 (1-3) | <0.001  <0.001  <0.001  <0.001  <0.001  <0.001 |
| QMVC peak (kg), median (IQR) | 30 (22-39) | 34 (27-43) | 30 (23-39) | 26 (20-36) | 25 (19-32) | <0.001 |

Values are given as the median and interquartile range (IQR), or No. of cases (%). Baseline data of 630 patients are included. P-values estimated using Wilcoxon-Mann-Whitney test for continuous data, and Chi-square test for categorical data.

FEV_1_ = forced expiratory volume in one second. GOLD = global initiative for obstructive lung disease. MRC = Medical Research Council. QMVC = quadriceps maximum voluntary contraction. SPPB = short physical performance battery. 4MGS = four-metre gait speed. 6MWT = six-minute walk test.

### **Table S6.** Adjusted multivariable associations, with the occurrence of death, by years of follow-up.

|  | **3 years (n = 60 deaths)** | | | | **5 years (n = 121 deaths)** | | | |
| --- | --- | --- | --- | --- | --- | --- | --- | --- |
| **Baseline Characteristics** | **Hazard ratio (95% CI) ^a^** | ***P* value ^a^** | **Hazard ratio (95% CI) ^b^** | ***P* value ^c^** | **Hazard ratio (95% CI) ^a^** | ***P* value ^a^** | **Hazard ratio (95% CI) ^b^** | ***P* value ^c^** |
| **Description** |  |  |  |  |  |  |  |  |
| Age – per 10 year increase | 1.42 (1.01 to 2.01) | 0.046 | 1.61 (1.13 to 2.30) | 0.008 | 1.54 (1.20 to 1.97) | 0.001 | 1.76 (1.36 to 2.28) | < 0.001 |
| Sex – male | 1.48 (0.83 to 2.63) | 0.186 | 1.53 (0.85 to 2.75) | 0.154 | 1.35 (0.91 to 2.02) | 0.138 | 1.36 (0.91 to 2.04) | 0.133 |
| Body mass index – per 1 point increase | 0.89 (0.84 to 0.94) | < 0.001 | 0.91 (0.86 to 0.97) | 0.002 | 0.92 (0.89 to 0.96) | < 0.001 | 0.94 (0.91 to 0.98) | 0.002 |
| **Lung function** |  |  |  |  |  |  |  |  |
| Smoking status – current | 2.02 (1.18 to 3.46) | 0.011 | 1.65 (0.96 to 2.86) | 0.072 | 1.77 (1.20 to 2.61) | 0.004 | 1.51 (1.02 to 2.25) | 0.040 |
| FEV_1_ – per 5% increase %predicted | 0.86 (0.79 to 0.94) | 0.001 | 0.93 (0.84 to 1.02) | 0.114 | 0.84 (0.79 to 0.89) | < 0.001 | 0.88 (0.82 to 0.95) | < 0.001 |
| MRC dyspnoea score – 2-4 | 1.44 (0.52 to 4.01) | 0.487 | 1.00 (0.35 to 2.86) | 0.999 | 1.93 (0.84 to 4.42) | 0.121 | 1.33 (0.57 to 3.09) | 0.507 |
| GOLD stage – per increase to next stage | 1.95 (1.36 to 2.80) | < 0.001 | 1.45 (0.95 to 2.22) | 0.086 | 1.83 (1.36 to 2.46) | < 0.001 | 1.83 (1.36 to 2.46) | < 0.001 |
| **Musculoskeletal measures** |  |  |  |  |  |  |  |  |
| Six-minute walk distance – per 30 metre increase | 0.87 (0.82 to 0.92) | < 0.001 | 0.85 (0.78 to 0.92) | < 0.001 | 0.89 (0.85 to 0.92) | < 0.001 | 0.88 (0.84 to 0.94) | < 0.001 |
| SPPB score (0-12) – per 1 point increase | 0.80 (0.71 to 0.89) | < 0.001 | 0.81 (0.72 to 0.92) | 0.002 | 0.93 (0.85 to 1.02) | 0.125 | 0.93 (0.85 to 1.02) | 0.125 |
| Functional limitation (SSPB) – yes | 2.13 (1.24 to 3.66) | 0.006 | 1.85 (1.04 to 3.28) | 0.036 | 1.33 (0.88 to 2.00) | 0.173 | 1.33 (0.88 to 2.00) | 0.173 |
| 4MGS score (0-4) – per point increase | 0.63 (0.48 to 0.84) | 0.002 | 0.67 (0.49 to 0.93) | 0.015 | 0.88 (0.69 to 1.13) | 0.310 | 0.88 (0.69 to 1.13) | 0.310 |
| Balance score (0-4) – per increase of 1 point | 0.63 (0.49 to 0.81) | < 0.001 | 0.63 (0.48 to 0.82) | 0.001 | 0.80 (0.65 to 0.99) | 0.040 | 0.80 (0.65 to 0.99) | 0.040 |
| Chair stand score (0-4) – per point increase | 0.79 (0.65 to 0.96) | 0.020 | 0.84 (0.68 to 1.04) | 0.112 | 0.96 (0.82 to 1.11) | 0.565 | 0.96 (0.82 to 1.11) | 0.565 |
| QMVC peak – per 1 kg increase | 0.95 (0.92 to 0.97) | < 0.001 | 0.97 (0.94 to 1.00) | 0.082 | 0.96 (0.94 to 0.98) | 0.111 | 0.98 (0.96 to 1.00) | 0.111 |

Total n = 630. Hazard ratios were estimated using Cox regression. All analyses were stratified by recruitment centre. Data after one-year follow-up are not included due to too few events (n = 15).

^a^ Adjusted for age and sex

^b^ Adjusted for age, sex, body mass index, smoking status, FEV_1_%, and MRC dyspnoea score

^c^ P values based on Cox regression.

CI = confidence intervals. FEV_1_ = forced expiratory volume in one second. GOLD = global initiative for obstructive lung disease. MRC = Medical Research Council. QMVC = quadriceps maximum voluntary contraction. SPPB = short physical performance battery. 4MGS = four-metre gait speed. 6MWT = six-minute walk test.

### **Table S7.** Cox proportional hazards regression analyses for all-cause mortality during follow-up, using composite scores for BODE indices.^a^

| **Variable** | Model 1: BMI | Model 2: BMI, MRC | Model 3: BMI, MRC, FEV_1_% | Model 4: BODE_6MWT_ | Model 5: BODE_SPPB_ | Model 6: BODE_4MGS_ | Model 7: BODE_BALANCE_ |
| --- | --- | --- | --- | --- | --- | --- | --- |
|  | **Hazard Ratio (95% CI)** | | | | | | |
| BMI – per 1 point increase | 0.90 (0.85 to 0.95) | 0.91 (0.86 to 0.95) | 0.91 (0.87 to 0.96) | ~ | ~ | ~ | ~ |
| MRC dyspnoea score | ~ | 1.38 (1.10 to 1.72) | 1.27 (0.99 to 1.63) | ~ | ~ | ~ | ~ |
| FEV_1_ – per 5% increase %predicted | ~ | ~ | 0.93 (0.85 to 1.02) | ~ | ~ | ~ | ~ |
| BODE | ~ | ~ | ~ | 1.30 (1.17 to 1.44) | 1.34 (1.19 to 1.51) | 1.35 (1.19 to 1.52) | 1.40 (1.23 to 1.58) |
| C-index | 0.608 (0.559 to 0.641) | 0.649 (0.608 to 0.679) | 0.649 (0.609 to 0.685) | 0.671 (0.641 to 0.693) | 0.667 (0.627 to 0.694) | 0.670 (0.634 to 0.694) | 0.682 (0.646 to 0.702) |
| Goodness of fit, chi2(3) | 0.22 | 5.17 | 7.91 | 0.75 | 3.88 | 3.08 | 5.43 |
| *P* > chi2 | 0.894 | 0.160 | 0.048 | 0.862 | 0.274 | 0.379 | 0.143 |
| Change in C-statistic | -0.063 (-0.101 to 0.033) | -0.021 (-0.044 to -0.001) | -0.021 (-0.050 to 0.010) | Reference | -0.003 (-0.018 to 0.012) | -0.001 (-0.013 to 0.014) | 0.011 (-0.003 to 0.027) |

^a^ All models were stratified by recruitment centre. BMI = body mass index. CI = confidence intervals. FEV_1_% = predicted forced expiratory volume 1 second. MRC = Medical Research Council. SPPB = short physical performance battery. 4MGS = four-metre gait speed. 6MWT = six-minute walk test.

### **Table S8.** Cox proportional hazards regression analyses for all-cause mortality, using continuous data after multiple imputation.

| **Model** | Model 1: BMI | Model 2: BMI, MRC | Model 3: BMI, MRC, FEV_1_% | Model 4: BODE_6MWT_ | Model 5: BODE_SPPB_ | Model 6: BODE_4MGS_ | Model 7: BODE_BALANCE_ |
| --- | --- | --- | --- | --- | --- | --- | --- |
| **Variable** | **Hazard Ratio (95% CI)** | | | | | | |
| BMI – per 1 point increase | 0.91 (0.87 to 0.96) | 0.92 (0.88 to 0.96) | 0.93 (0.88 to 0.97) | ~ | ~ | ~ | ~ |
| MRC dyspnoea score | ~ | 1.42 (1.16 to 1.74) | 1.31 (1.04 to 1.63) | ~ | ~ | ~ | ~ |
| FEV_1_ – per 5% increase %predicted | ~ | ~ | 0.99 (0.97 to 1.00) | ~ | ~ | ~ | ~ |
| BODE | ~ | ~ | ~ | 1.29 (1.17 to 1.42) | 1.33 (1.19 to 1.48) | 1.33 (1.20 to 1.49) | 1.37 (1.22 to 1.53) |
| C-index | 0.546 (0.529 to 0.560) | 0.617 (0.606 to 0.629) | 0.662 (0.650 to 0.673) | 0.690 (0.679 to 0.699) | 0.661 (0.650 to 0.671) | 0.660 (0.650 to 0.672) | 0.673 (0.658 to 0.685) |

Total n = 630. All models were stratified by recruitment centre. CI = confidence intervals. BMI = body mass index. MRC = Medical Research Council. FEV_1_% = predicted forced expiratory volume one second. SPPB = short physical performance battery. 4MGS = four-metre gait speed. 6MWT = six-minute walk test.

### **Table S9.** Risk indices using point system, by survival status.

| Risk indices | Median (IQR) | Survivors | Non-survivors | *P* value † |
| --- | --- | --- | --- | --- |
| BODE_6MWT_ (0-10) | 3 (1-5) | 3 (1-5) | 5 (2-7) | < 0.001 |
| BODE_SPPB_ (0-10) | 3 (1-4) | 2 (1-4) | 4 (2-6) | < 0.001 |
| BODE_4MGS_ (0-10) | 3 (1-4) | 2 (1-4) | 4 (2-6) | < 0.001 |
| BODE_BALANCE_ (0-10) | 2 (1-4) | 2 (1-4) | 4 (2-6) | < 0.001 |

† Wilcoxon-Mann-Whitney test

SPPB = short physical performance battery. 4MGS = four-metre gait speed. 6MWT = six-minute walk test.

### **Table S10.** Risk indices using point system, by cause of death.

| Risk indices | Pulmonary | Cardiac | Cancer | Other | *P* value † |
| --- | --- | --- | --- | --- | --- |
| BODE_6MWT_ (0-10) | 6 (4-7) | 2 (1-7) | 2 (1-4) | 3 (1-7) | 0.004 |
| BODE_SPPB_ (0-10) | 5 (3-6) | 2 (1-5) | 2 (1-4) | 4 (2-6) | 0.004 |
| BODE_4MGS_ (0-10) | 6 (3-6) | 2 (1-5) | 2 (2-3) | 3 (2-6) | < 0.001 |
| BODE_BALANCE_ (0-10) | 5 (3-6) | 4 (1-6) | 3 (1-3) | 4 (2-5) | 0.007 |

† Kruskal-Wallis equality-of-populations rank test

SPPB = short physical performance battery. 4MGS = four-metre gait speed. 6MWT = six-minute walk test.

## Supplementary Figures

Individuals screened (n = 746)

Study entry

Did not meet inclusion criteria (n = 12)

Screening

Individuals entered into study (n = 734)

Missing FEV_1_ / FVC ratio (n = 5)

Individuals completed visit (n = 729)

Baseline visit

Not flagged by NHS (n = 15)

Individuals with mortality status (n = 714)

Flagging

Missing biomarker data (n = 84)

- Body mass index (n = 7)

- Smoking status (n = 4)

- MRC dyspnoea score (n = 5)

- 6MW (n = 34)

- SPPB score (n = 8)

- QMVC (n = 27)

Individuals included in analysis (n = 630)

Analysis

Dead (n = 60)

Alive (n = 570)

FUP 3-yrs

Sensitivity analysis

Individuals with mortality status (n = 714). Missing data were imputed.

Dead (n = 71)

Alive (n = 643)

FUP 3-yrs

### **Figure S1.** Participant enrolment flow diagram with three years follow up of mortality.

Total number of deaths (n = 60). FEV_1_ = forced expiratory volume 1 second. FVC = forced vital capacity. FUP = follow-up period. MRC = Medical Research Council. NHS = National Health Services. QMVC = quadriceps maximum voluntary contraction. SPPB = short physical performance battery. 6MWT = six-minute walk test.

### **Figure S2.** Forest plot displaying standardized adjusted hazard ratios, by years of follow-up.

Hazard ratios were estimated using Cox regression. All analyses were adjusted for age, sex, body mass index, smoking status, FEV_1_%, and MRC dyspnoea score. Hazard ratios displayed are after 3 years of follow-up. Number of deaths was 15 after 1 year, 60 after 3 years, and 121 after 5 years of follow-up.

SD = standard deviation, CI = confidence intervals, BMI = body mass index, FEV_1_% = predicted forced expiratory volume 1 second, MRC = Medical Research Council, GOLD = global initiative for chronic obstructive lung disease, 6MWT = six-minute walk test, SPPB = short physical performance battery, 4MGS = 4-metre gait speed, QMVC = quadriceps maximum voluntary contraction.

### **Figure S3.** Change in C-index of BODE with additional components.

CI = confidence interval.

### **Figure S4.** Predicted and observed mortality by risk quartiles with associated 95% confidence intervals.

Total n = 630. X-axis indicates four risk groups with predicted mortality ranges. Y-axis indicates 3-year risk for mortality as a probability. (A) BODE_6MWT_, (B) BODE_SPPB_, (C) BODE_4MGS_, and (D) BODE_BALANCE_.

### **Figure S5.** Change scores C-index. Individual components BODE.

BMI = body mass index. MRC = Medical Research Council. 6MW = six-minute walk test. CI = confidence interval.

### **Figure S6.** Missing data patterns.

Six-minute walking distance is the most common missing variable (4%), followed by quadriceps maximum voluntary contraction (3%), and sniff nasal inspiratory pressure (2%). One percentage has missingness in these three variables. bmi = body mass index, fev1 = forced expiratory volume 1 second, fev1perc = predicted forced expiratory volume 1 second, mrc = Medical Research Council, qmvc = quadriceps maximum voluntary contraction.

### **Figure S7.** Boxplots, after multiple imputation.

### **Figure S8.** Kernel density plots, after multiple imputation.

### **Figure S9.** Diagnostic histograms, after multiple imputation. Examples of first imputed dataset only.

### **Figure S10.** Correlation matrix: Associations among the variables measured at baseline.

MRC = Medical Research Council dyspnoea scale. BMI = body mass index. Smoked = smoking status. FEVpct = forced expiratory volume in one second %predicted. SNIP = sniff nasal inspiratory pressure. Bal = balance (SPPB). QMVC = quadriceps maximum voluntary contraction. Gait = four-metre gait speed. Chair = chair stand (SPPB). DIST = six-minute walk distance. SPPB = short physical performance battery.

Correlation coefficients with a values <0.30 are considered negligible, 0.30 - 0.50 as moderate, and >0.50 as strong.^[[18]](#footnote-18)^

### **Figure S11.** Missing data patterns.

Margin plots in blue indicate the distribution of observed data given the other variable is observed. Red box plots indicate data distribution of the observed data given the other variable is missing. These plots indicate walking distance to be missing for those with lower QMVC scores, higher BMI, worse MRC dyspnoea scores, and worse FEV_1_. These plots indicate the potential for missingness not at random (MNAR). 6MW = six-minute walk. BMI = body mass index. FEV_1_ = forced expiratory volume in one second. QMVC = quadriceps maximum voluntary contraction. SNIP = sniff nasal inspiratory pressure.

1. Vestbo J, Anderson W, Coxson HO, Crim C, Dawber F, Edwards L, et al. Evaluation of COPD Longitudinally to Identify Predictive Surrogate End-points (ECLIPSE). Eur Respir J. 2008;31(4):869-73. doi: 10.1183/09031936.00111707. [↑](#footnote-ref-1)
2. Gale NS, Albarrati AM, Munnery MM, Munnery IC, Irfan M, Bolton CE, et al. Assessment of Risk in Chronic Airways Disease Evaluation (ARCADE): Protocol and preliminary data. Chron Respir Dis. 2014;11(4):199-207. doi: 10.1177/1479972314546765. [↑](#footnote-ref-2)
3. Mohan D, Gale NS, McEniery CM, Bolton CE, Cockcroft JR, MacNee W, et al. Evaluating the role of inflammation in chronic airways disease: the ERICA study. COPD. 2014;11(5):552-9. doi: 10.3109/15412555.2014.898031 [↑](#footnote-ref-3)
4. The International Classification of adult underweight, overweight and obesity according to BMI [Internet]. WHO. 2016 [cited 12 August 2016]. Available from: http://apps.who.int/bmi/index.jsp?introPage=intro_3.html. [↑](#footnote-ref-4)
5. Global Initiative for Chronic Obstructive Lung Disease (GOLD). Global Strategy for the Diagnosis, Management and Prevention of COPD. 2016. [↑](#footnote-ref-5)
6. Pavasini R, Guralnik J, Brown JC, di Bari M, Cesari M, Landi F, et al. Short Physical Performance Battery and all-cause mortality: systematic review and meta-analysis. BMC Med. 2016;14(1):215. doi: 10.1186/s12916-016-0763-7. [↑](#footnote-ref-6)
7. Bernabeu-Mora R, Medina-Mirapeix F, Llamazares-Herran E. The Short Physical Performance Battery is a discriminative tool for identifying patients with COPD at risk of disability (vol 10, pg 2619, 2015). Int J Chronic Obstr. 2016;11:623-. doi: 10.2147/Copd.S106593. [↑](#footnote-ref-7)
8. Celli BR, Cote CG, Marin JM, Casanova C, Montes de Oca M, Mendez RA, et al. The body-mass index, airflow obstruction, dyspnea, and exercise capacity index in chronic obstructive pulmonary disease. The New England journal of medicine. 2004;350(10):1005-12. doi: 10.1056/NEJMoa021322. [↑](#footnote-ref-8)
9. Esteban C, Quintana JM, Aburto M, Moraza J, Capelastegui A. A simple score for assessing stable chronic obstructive pulmonary disease. Qjm. 2006;99(11):751-9. doi: 10.1093/qjmed/hcl110. [↑](#footnote-ref-9)
10. Cote CG, Pinto-Plata VM, Marin JM, Nekach H, Dordelly LJ, Celli BR. The modified BODE index: validation with mortality in COPD. Eur Respir J. 2008;32(5):1269-74. doi: 10.1183/09031936.00138507. [↑](#footnote-ref-10)
11. Briggs A, Spencer M, Wang H, Mannino D, Sin DD. Development and validation of a prognostic index for health outcomes in chronic obstructive pulmonary disease. Arch Intern Med. 2008;168(1):71-9. doi: 10.1001/archinternmed.2007.37. [↑](#footnote-ref-11)
12. Kostianev SS, Hodgev VA, Iluchev DH. Multidimensional system for assessment of COPD patients. Comparison with BODE index. Folia Med (Plovdiv). 2008;50(4):29-38. [↑](#footnote-ref-12)
13. Puhan MA, Garcia-Aymerich J, Frey M, ter Riet G, Anto JM, Agusti AG, et al. Expansion of the prognostic assessment of patients with chronic obstructive pulmonary disease: the updated BODE index and the ADO index. Lancet. 2009;374(9691):704-11. doi: 10.1016/S0140-6736(09)61301-5. [↑](#footnote-ref-13)
14. Soler-Cataluna JJ, Martinez-Garcia MA, Sanchez LS, Tordera MP, Sanchez PR. Severe exacerbations and BODE index: two independent risk factors for death in male COPD patients. Respir Med. 2009;103(5):692-9. doi: 10.1016/j.rmed.2008.12.005. [↑](#footnote-ref-14)
15. Mehrotra N, Freire AX, Bauer DC, Harris TB, Newman AB, Kritchevsky SB, et al. Predictors of mortality in elderly subjects with obstructive airway disease: the PILE score. Ann Epidemiol. 2010;20(3):223-32. doi: 10.1016/j.annepidem.2009.11.005. [↑](#footnote-ref-15)
16. Celli BR, Locantore N, Yates J, Tal-Singer R, Miller BE, Bakke P, et al. Inflammatory biomarkers improve clinical prediction of mortality in chronic obstructive pulmonary disease. Am J Respir Crit Care Med. 2012;185(10):1065-72. doi: 10.1164/rccm.201110-1792OC. [↑](#footnote-ref-16)
17. Stolz D, Kostikas K, Blasi F, Boersma W, Milenkovic B, Lacoma A, et al. Adrenomedullin refines mortality prediction by the BODE index in COPD: the "BODE-A" index. Eur Respir J. 2014;43(2):397-408. doi: 10.1183/09031936.00058713. [↑](#footnote-ref-17)
18. Cohen J. Statistical Power Analysis for the Behavioral Sciences: Elsevier Science; 2013 [↑](#footnote-ref-18)
